# Benefits of Repetitive Transcranial Magnetic Stimulation (rTMS) for Spastic Subjects: Clinical, Functional, and Biomechanical Parameters for Lower Limb and Walking in Five Hemiparetic Patients

**DOI:** 10.1155/2014/389350

**Published:** 2014-04-29

**Authors:** Luc Terreaux, Raphael Gross, Fabien Leboeuf, Hubert Desal, Olivier Hamel, Jean Paul Nguyen, Chantal Pérot, Kévin Buffenoir

**Affiliations:** ^1^Department of Neurosurgery and Neurotraumatology, CHU de Nantes, 1, place Alexis Ricordeau, 44093 Nantes, France; ^2^UMR CNRS 7338 Biomécanique et Bioingénierie, Université de Technologies de Compiègne, BP 20529, 60205 Compiègne, France; ^3^Movement Analysis Laboratory, Department of Physical Medicine and Rehabilitation, Hôpital Saint Jacques, CHU Nantes, 1, place Alexis Ricordeau, 44093 Nantes, France; ^4^Department of Neuroradiology, CHU de Nantes, 1, place Alexis Ricordeau, 44093 Nantes, France; ^5^INSERM EA3826, “Pain, Neuromodulation, and Quality of Life”, CHU de Nantes, 1, place Alexis Ricordeau, 44093 Nantes, France

## Abstract

*Introduction.* Spasticity is a disabling symptom resulting from reorganization of spinal reflexes no longer inhibited by supraspinal control. Several studies have demonstrated interest in repetitive transcranial magnetic stimulation in spastic patients. We conducted a prospective, randomized, double-blind crossover study on five spastic hemiparetic patients to determine whether this type of stimulation of the premotor cortex can provide a clinical benefit. *Material and Methods.* Two stimulation frequencies (1 Hz and 10 Hz) were tested versus placebo. Patients were assessed clinically, by quantitative analysis of walking and measurement of neuromechanical parameters (*H* and *T* reflexes, musculoarticular stiffness of the ankle). *Results.* No change was observed after placebo and 10 Hz protocols. Clinical parameters were not significantly modified after 1 Hz stimulation, apart from a tendency towards improved recruitment of antagonist muscles on the Fügl-Meyer scale. Only cadence and recurvatum were significantly modified on quantitative analysis of walking. Neuromechanical parameters were modified with significant decreases in *H*_max⁡_ /*M*_max⁡_ and *T*/*M*_max⁡_ ratios and stiffness indices 9 days or 31 days after initiation of TMS. *Conclusion.* This preliminary study supports the efficacy of low-frequency TMS to reduce reflex excitability and stiffness of ankle plantar flexors, while clinical signs of spasticity were not significantly modified.

## 1. Introduction

Any central nervous system lesion induces major reorganization of partially denervated underlying structures. The definition of spasticity, resulting from reorganization of spinal segmental reflexes, which no longer subjected to supraspinal control, was established by Lance [1] and was recently modified by the EU-SPASM consortium: [2] “*Spasticity-disordered sensorimotor control, resulting from an upper motor neuron lesion, presenting as intermittent or sustained involuntary activation of muscles.*” The prevalence of spasticity varies according to the type of brain lesion: more than 90% in cerebral palsy [3], almost 50% after head injury [4], 37 to 78% in multiple sclerosis (MS) [5, 6], and 19 to 35% after stroke [7]. Spasticity predominantly affects the extensor, postural, and antigravity muscles of the lower limbs [8, 9]. Spasticity of the foot results in equinus deformity due to overactivity of triceps surae and varus deformity due to overactivity of inversion muscles and weakness of eversion muscles. All these deformities are responsible for unstable gait support, with or without pain, poor clearance of the foot during the swing phase, difficulty wearing shoes, and limitation of walking distance.

The cerebral cortex exerts a powerful inhibitory effect on muscle tone, mediated via the reticular formation, as the ventromedial medullary reticular formation decreases muscle tone with a facilitating effect of the premotor cortex [8]. On the basis of these findings, repetitive transcranial magnetic stimulation (rTMS) must therefore be targeted to the premotor cortex in order to reinforce suprasegmental inhibition and consequently decrease spasticity.

Three published trials have demonstrated interesting effects of rTMS on spastic symptoms using various frequencies and various targets, on *H* reflexes in healthy subjects [10], spasticity in MS patients [11], and decreased spasticity in combination with neurological recovery [12].

We therefore conducted a prospective, randomized, double-blind study of the effects of rTMS on clinical, functional, and neuromechanical parameters in patients with disabling spastic hemiplegia.

## 2. Material and Methods

### 2.1. Population

Five patients (5 men with a mean age of 56 years [46–75 years]) with spastic hemiparesis were consecutively included in this study by a multidisciplinary “spasticity” clinic. These 5 patients presented left-sided hemiparesis. Spastic hemiparesis was secondary to stroke in 4 patients (hemorrhagic stroke in 3 cases and ischemic stroke in 1 case) and intracranial tumour surgery (convexity meningioma, right central sulcus) in 1 case.

### 2.2. Stimulation Protocol and Parameters

According to a number of animal studies, the premotor cortex is a powerful regulator of contralateral muscle tone [13, 14]. The premotor cortex, integrating extrapyramidal pathways, is situated, according to the cytoarchitectural studies of Brodmann (Area 6) followed by Vogt (Area 6*αβ*), at the intersection of the superior frontal sulcus and the precentral gyrus [15]. This area appears to facilitate activation of dorsal reticulospinal pathways located in the ventromedial medullary reticular formation [16] and projecting onto the spinal cord via a dorsal reticulospinal tract, which inhibit muscle tone. The objective of this study was therefore to modulate this cortical regulation of spinal reflex activity, the suppression of which, following a supraspinal lesion, constitutes the pathophysiological basis for spasticity.

Each of the stimulation protocols was targeted to the premotor cortex on the side of the lesion (i.e, the hemisphere contralateral to the side of spastic hemiparesis). According to the literature, the premotor cortex would appear to be the most promising target in this indication [11, 12]. This zone was identified by neuronavigation based on acquisition of a 3D T1 MRI sequence for each patient. Anatomical correspondence was performed according to the manufacturer's recommendations (Nexstim): use of glasses in which spatial positioning is detected by triangulation by means of an infrared camera, followed by palpation of landmarks on the skull surface by a cursor also localized in space. The error estimated by software was verified (refusal of an error ≥2 mm).

**Figure 1:**
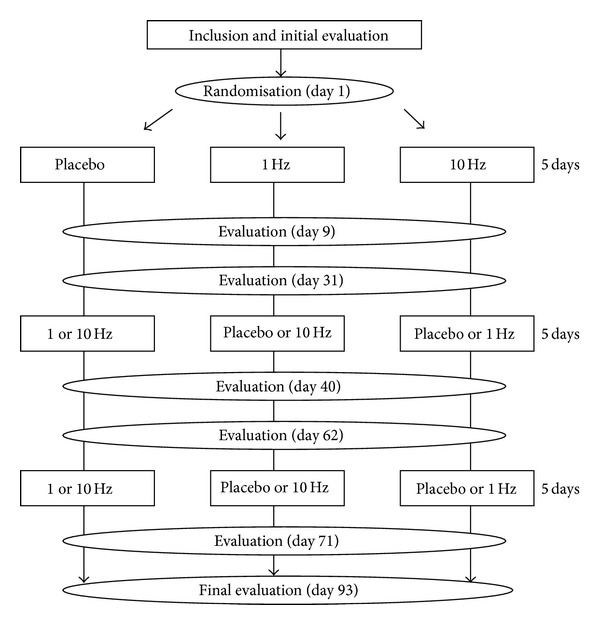
Design of the study.

This prospective, randomized, crossover study was conducted under double-blind conditions (blinded person performing stimulation and randomization of the stimulation protocols). Each patient received the 3 stimulation protocols (Figure 1), including a placebo protocol, in random order, using a MagProR30 stimulator in compliance with the TMS Safety Consensus Group guidelines [17]. Each daily stimulation phase lasted 16 minutes and 40 seconds, that is, 1,000 impulses, for five consecutive days. Stimulation weeks were separated by an interval of at least one month and a complete assessment was performed prior to each session (D0). These assessments were repeated 4 days and 21 days (D9 then D31) after the end of stimulation.

The first protocol consisted of continuous low frequency 1 Hz stimulation at 90% of the motor threshold and the second protocol consisted of high frequency 10 Hz stimulation at 100% of the motor threshold, composed of twenty 5-second trains separated by 45-second intervals. We chose these two different frequencies according to the literature [11, 12]. Placebo stimulation was randomized to one of the two frequencies with application of an audio coil, not delivering any magnetic impulses.

### 2.3. Clinical Assessment

#### 2.3.1. Spasticity

Ankle spasticity, the primary endpoint of the study, was scored by the modified Ashworth scale [18], with the knee flexed and the knee extended, at slow speed then at rapid speed.

#### 2.3.2. Neurological Assessment

Residual antagonist muscle activity was evaluated in the supine position by the simple order: “raise your foot.” The percentage amplitude of recruitment of antagonists was evaluated: less than 25%, between 25% and 50%, between 50% and 75%, or between 75% and 100% (score from 1 to 4). Fine touch on the leg and foot and proprioception of the big toe were tested with the patient's eyes closed. Sensory assessment was scored as normal, decreased, or absent. Pain when wearing shoes was assessed by a visual analogue scale (VAS) from 0 to 10, where 0 represented no pain and 10 represented the worst imaginable pain. Finally, the global neurological recovery score was evaluated by the Fügl-Meyer scale [19, 20].

### 2.4. Analysis of Walking

**Figure 2:**
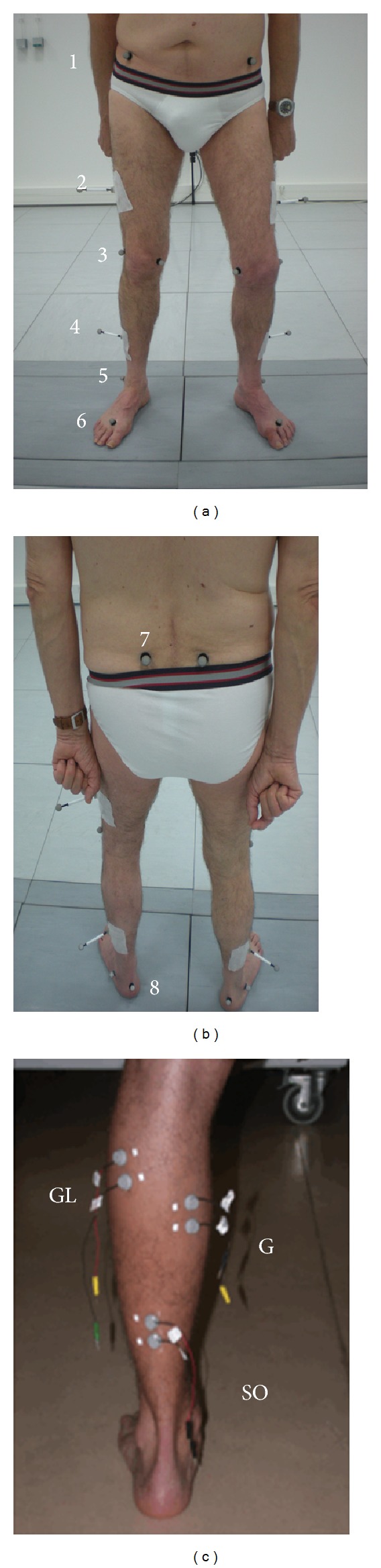
Anterior (a) and posterior (b) views of optoelectronic markers and positioning of electrodes (c) on the 3 heads of triceps surae for recording of electromyographic responses *M*, *H*, and *T*. (1) Anterior superior iliac spine; (2) thigh marker; (3) lateral femoral condyle; (4) leg marker; (5) lateral malleolus; (6) head of second metatarsus; (7) posterior superior iliac spine; (8) calcaneus. G: medial gastrocnemius; GL: lateral gastrocnemius; SO: soleus.

Quantitative analysis of walking (QAW), performed in the movement analysis laboratory, allows objective and systematic acquisition of spatiotemporal and kinematic parameters [21]. Acquisition and data processing systems and the positioning of markers on the patient's lower limbs have been previously described by our laboratory [22]. Photographs of the position of the markers were taken during first application of the markers to decrease the risk of subsequent marker placement errors (Figures 2(a) and 2(b)). A static acquisition was obtained by recognition of the markers using 6 infrared cameras (Vicon MX F 40). Dynamic acquisitions were obtained during spontaneous walking, on a maximum of 10 passages, with bare feet in every case and without assistance whenever possible. Conditions were identical between the various sessions to allow objective comparison of all parameters.

#### 2.4.1. Spatiotemporal Parameters

The following parameters, averaged over several cycles, were recorded for each assessment: gait velocity of the affected limb (m/s), cadence (steps/min), stride length (m), and percentages of single limb and double limb support.

#### 2.4.2. Kinematic Parameters

The position of the knee in maximum extension was quantified to detect possible recurvatum deformity. The angle of dorsiflexion at the strike phase, maximum dorsiflexion during the stance phase and at the beginning of the swing phase, and maximum plantar flexion at the end of the swing phase was measured in the sagittal plane of the ankle. The range of ankle motion was also measured.

### 2.5. Electromyographic Assessment

*H* and *T* reflexes and maximum motor response (*M*_max⁡_) were recorded for each of the three muscle heads of triceps surae, according to a previously described technique [23]. Positioning of recording electrodes is illustrated in Figure 2(c). Maximum peak-to-peak amplitudes of the various responses were measured and averaged on 10 recordings (*H*_max⁡_, *M*_max⁡_, and *T*), and *H*_max⁡_/*M*_max⁡_ and *T*/*M*_max⁡_ ratios were calculated. The duration of *H* or *M* responses and their latencies were also calculated for each response by manually selecting the start and end of each response.

### 2.6. Assessment of Passive Stiffness of the Ankle

Passive stiffness of the ankle was evaluated by using an ankle ergometric device designed by the Université de Technologie de Compiègne [24] and by applying sinusoidal perturbations with an amplitude of ±1.5 on either side of the ankle neutral position to the ankle at rest (90). The frequency of sinusoidal perturbations ranged from 4 to 16 Hz with increments of 1 Hz. Application of sinusoidal perturbations induced variations of passive torque of the ankle joint, the amplitude and dephasing of which are frequency-dependent. A stiffness constant (Kp, expressed in Nm/rad), reflecting passive musculoarticular stiffness of the ankle, was calculated from Bode diagrams, as described by Lambertz et al. [25].

### 2.7. Ethics Committee Approval

The study design and all investigations were validated by Nantes University Hospital, the study sponsor. The* Comité de Protection des Personnes Ouest V* (IEC/IRB) issued a positive opinion to conduct the study. The* Agence Française de Sécurité Sanitaire des Produits de Santé* (French Health Products Safety Agency) also authorised the submitted project. All patients were informed and signed an informed consent form.

### 2.8. Data Processing and Statistical Methods

Ergometric data were analysed by Matlab (The MathWorks, Inc., Natick, MY 01760-2098, USA) using Synerg software developed in the laboratory. EMG data were recorded by Systemg software and were analysed by Neuromecanik software. This software was developed in the Compiègne laboratory. Statistical analyses were performed with JMP 9.0.2 software (SAS Institute INC 2010). A paired* t*-test was used for all comparisons of means. The limit of significance was 0.05.

## 3. Results

The mean interval between the initial accident responsible for spasticity and initiation of the protocol was 79 months [range: 29–136 months]. The five patients complied with all aspects of the protocol and the assessments. No serious adverse event was observed. Two patients experienced transient headache that resolved in response to treatment with step I analgesics. One patient reported tiredness related to travelling to and from the hospital for the daily visits during the week of stimulation. No significant difference was observed for any of the parameters studied before each stimulation session, which validates the reproducibility of measurements and return to baseline before each new stimulation protocol.

No statistically significant change or tendency to change was observed for any of the clinical, neuromechanical, and gait parameters after the placebo and 10 Hz stimulation protocols. Significant changes were demonstrated after the 1 Hz stimulation protocol. This section will therefore be confined to a detailed description of these changes.

### 3.1. Clinical Assessment

The main clinical findings are summarized in Table 1.

#### 3.1.1. Spasticity

All patients initially presented equinovarus deformity of the foot. No modification of this deformity was observed at the various assessments after the stimulation protocols. No modification of the Ashworth score was observed at the various clinical assessments performed during this stimulation protocol, regardless of the test conditions.

#### 3.1.2. Neurological Assessment

Clinical examination did not reveal any changes in the motor function of the peroneal and triceps surae muscles. In contrast, a tendency towards improved recruitment of antagonist muscles was observed on D9, especially for the maximum percentage angulation of dorsiflexors under active conditions. This tendency was amplified and became significant on D31 (Table 1: from 1 on D0 to 1.8 on D31,* t* -test, *P* = 0.049).

No significant difference was observed for touch sensation, but a tendency towards improvement of proprioception was observed: 3 of the patients in whom proprioception was absent on D0 presented decreased but present proprioception on D9 and D31. One patient reported pain when wearing shoes (VAS: 4) at the baseline assessment. Stimulation suppressed this pain in this patient (VAS: 0 on D9 and D31).

The mean Fügl-Meyer score was 28 at the baseline assessment and was significantly increased at the D9 assessment (Table 1,* t*-test, *P* = 0.047) with return to baseline on D31.

### 3.2. Walking Analysis

All parameters recorded before stimulation (D0) and on the early (D9) and late (D31) assessments after the 1 Hz and 10 Hz stimulation protocols are presented in Table 2.

#### 3.2.1. Spatiotemporal Parameters

Gait velocity, stride length, and stance phase/swing phase distribution remained unchanged at the various assessments. Cadence significantly decreased between D0 and D9 (*t*-test, *P* = 0.0117) and between D0 and D31 (*P* = 0.0424).

#### 3.2.2. Kinematic Parameters

Prior to any therapeutic intervention, spastic patients presented an equinus strike phase (mean defect of ankle dorsiflexion of −8.91°). Ankle dorsiflexion remained extremely limited throughout gait (Table 2). Three patients presented recurvatum of the knee (reflected by positive maximum knee extension values). This parameter was significantly improved at the long-term assessment (Table 2,* t*-test, *P* = 0.042), as recurvatum was no longer observed in these 3 patients.

### 3.3. Electrophysiological Assessment

Electrophysiological parameters varied in an identical way for the 3 heads of triceps surae. In view of the predominant role of the soleus muscle in the spastic equine foot phenomenon, only the variations observed in this muscle are presented here. No significant difference was demonstrated for the mean values of latency and duration of the various responses on all of the measurements performed on D0, D9, and D31.

Mean *H*_max⁡_/*M*_max⁡_ and *T*/*M*_max⁡_ ratios were significantly decreased at the two assessments after the 1 Hz protocol (Table 3): 30% reduction of the mean *H*_max⁡_/*M*_max⁡_ ratio (*t*-test, *P* = 0.0295) and 33% reduction of the mean *T*/*M*_max⁡_ ratio (*t*-test, *P* = 0.0006) on D9. These reductions remained significant between D0 and D31: 35% reduction of the mean *H*_max⁡_/*M*_max⁡_ ratio (*t*-test, *P* = 0.0394) and 33% reduction of the mean *T*/*M*_max⁡_ ratio (*t*-test, *P* = 0.0168).

### 3.4. Assessment of Passive Stiffness of the Ankle

A slight but significant reduction of passive stiffness of the ankle was observed on D9 after 1 Hz stimulation (Table 3,* t*-test, *P* = 0.039). This reduction remained stable on D31.

## 4. Discussion

This study based on a series of 5 patients is the first randomized, double-blind, crossover study to assess all clinical and complementary parameters of spasticity. The main limitation of this study is the small number of patients, as the main limiting factor for recruitment is the cost of transport of these patients who cannot travel unassisted (the study required a total of 25 trips to and from the hospital for each patient).

This prospective study did not reveal any modification of spastic symptoms regardless of the stimulation protocol tested, as no variation of the Ashworth score (always evaluated by the same clinician) or presentation of the foot (no variation of the equinovarus deformity after stimulation) was demonstrated. Two studies [11, 12] assessed the clinical parameters of spasticity and their changes after treatment by rTMS. The first study [11] concerned a population of patients with multiple sclerosis complicated by spasticity but did not provide any details concerning the spinal or cerebral site of multiple sclerosis. The stimulation target differed from that adopted in the present study, as these authors performed primary motor cortex stimulation. The duration of stimulation was also longer (2 weeks) than in our protocol. No modification of clinical parameters (the only parameters evaluated in this study) was observed after 1 Hz stimulation. However, an average 2-point reduction of the global Ashworth score (corresponding to the sum of modified Ashworth scores evaluated in the ankle, knee, and hip) was demonstrated immediately after and one week after the stimulation protocol. The second study published in the literature [12] simultaneously evaluated neurological recovery and improvement of spasticity in response to a low frequency (1 Hz) premotor cortex stimulation protocol in stroke patients. A more marked improvement of spasticity was observed after stimulation of the contralateral side to the spastic hemiparesis, but this study reported few details concerning clinical parameters and used various scores that are not validated in the literature. Overall, rTMS therefore appears to have only a minor effect on spasticity* per se*, regardless of the stimulation parameters and targets tested.

The 1 Hz stimulation protocol of the premotor cortex ipsilateral to the lesion appeared to have a positive effect on antagonist muscle recruitment (significant facilitation of dorsiflexion of the ankle 3 weeks after completion of the stimulation protocol) and on the functional capacities of the lower limb (increased Fügl-Meyer score). These results are supported by the increased range of motion of the ankle demonstrated during quantitative analysis of walking. However, this comparison must be interpreted very cautiously, as it is based on two different types of recruitment: active recruitment tested during clinical examination and reflex recruitment during walking. Moreover, no improvement of gait was demonstrated on quantitative analysis regardless of the type of stimulation. On the contrary, a tendency towards slowing of gait was observed after stimulation, very probably related to “destabilization” of the patient in relation to the changes of two kinematic parameters observed during this study (range of motion of the ankle and decreased recurvatum of the knee). Adaptation of gait to these modifications is obviously a long process and the effect of stimulation was so short-lived that the patient did not have time to modify gait in order to derive a benefit from these improved parameters. This aspect will need to be assessed in longer term studies of repetitive stimulation protocols. An improvement of gait would probably be observed after several repetitions of the same 1 Hz rTMS protocol.

The modifications of neuromechanical parameters of the ankle observed in this study appear to be more promising, as the 1 Hz stimulation protocol clearly appeared to reinforce suprasegmental inhibition (greater than 30% reduction of the *H*_max⁡_/*M*_max⁡_ and *T*/*M*_max⁡_ ratios), while 10 Hz and placebo protocols did not induce any modification of these parameters. Similar results were reported in a study conducted on 10 healthy subjects after a 5 Hz primary motor cortex stimulation protocol [10]. However, this result is in complete contradiction to the effect reported in the literature in patients with multiple sclerosis [11], as a reduction of this *H*_max⁡_/*M*_max⁡_ ratio was demonstrated after the 10 Hz stimulation protocol, which was not observed in the present study. This previous study also reported a tendency towards an increase of this ratio in healthy subjects after the 1 Hz stimulation protocol. Reduction of the *H*_max⁡_/*M*_max⁡_ ratio in our series is supported by the reduction of the *T*/*M*_max⁡_ ratio, which was more marked at the early assessment, suggesting a complementary action of rTMS on elements of the T reflex loop not involved in the *H* reflex loop (spindle sensitivity, gamma drive, and spindle recruitment by more compliant elastic structures) [26], correlated with the reduction of passive stiffness of the ankle observed in this study. Testing of ankle stiffness comprises a passive component (particularly the length and elasticity of muscle and tendon structures) and an active component (spindle sensitivity, gamma drive, and spindle recruitment). One hypothesis to explain this reduction of passive stiffness would be a reduction of muscle tone and therefore the number of residual cross-bridges, thereby making the muscle more compliant. This effect on the muscle tone would have consequences on both the passive and active components of ankle stiffness. Further investigations must be conducted on these aspects. This effect on ankle stiffness remained stable at the long-term assessment (stability of stiffness and *T*/*M*_max⁡_ ratio), while the effect of rTMS on suprasegmental inhibition was reinforced (more marked reduction of the *H*_max⁡_/*M*_max⁡_ ratio at the long-term assessment).

## 5. Conclusion

In summary, the only rTMS protocol with an objective effect in this study was the low frequency (1 Hz) protocol targeted to the premotor cortex on the side of the lesion. Only a moderate clinical effect was observed, predominantly on the consequences of spasticity (recruitment of antagonists and recurvatum) but not on spastic symptoms* per se*. The observed effect appeared to be due to reinforcement of suprasegmental inhibition acting on the *H* reflex loop and a reduction of muscle tone, but these mechanisms need to be confirmed. A long-term repetitive stimulation protocol now needs to be proposed to enable the patient to adapt his gait to these changes, which would therefore probably facilitate walking, which remains the primary objective of any therapeutic intervention designed to improve spasticity.

## Figures and Tables

**Table 1:** Course of clinical parameters (mean ± standard deviation) during the 1 Hz and 10 Hz († brackets) stimulation protocols: before (D0) and after (D9: early assessment; D31: late assessment).

Parameter	D0	D9	D31
Modified Ashworth Knee flexed/rapid speed	2.0 ± 0.9	2.0 ± 0.9 [2.0 ± 0.9]^†^	2.0 ± 1.1 [2.0 ± 1.0]^†^
Modified Ashworth Knee extended/rapid speed	2.2 ± 0.8	2.2 ± 0.8 [2.2 ± 0.8]^†^	2.2 ± 0.8 [2.2 ± 0.8]^†^
Recruitment of antagonists	1.0 ± 0.8	1.3 ± 1.3 [1.2 ± 1.3]^†^	1.8 ± 1.7* [1.3 ± 1.1]^†^
Fügl-Meyer score in the lower limb	28.0 ± 8.8	29.5 ± 9.0* [28.4 ± 8.9]^†^	28.5 ± 9.3 [29.0 ± 8.7]^†^

*Statistically significant difference (*t*-test, *P* < 0.05).

**Table 2:** Course of parameters recorded during quantitative analysis of walking (mean ± standard deviation) during the 1 Hz and 10 Hz († brackets) stimulation protocols: before (D0) and after (D9: early assessment; D31: late assessment).

Parameter	D0	D9	D31
Gait velocity (m/s)	0.48 ± 0.46	0.46 ± 0.43 [0.47 ± 0.40]^†^	0.44 ± 0.41 [0.45 ± 0.42]^†^
Cadence (steps/min)	40.19 ± 10.76	38.69 ± 10.54* [38.84 ± 9.57]^†^	38.03 ± 10.95* [37.87 ± 9.91]^†^
Stride length (m)	0.62 ± 0.48	0.63 ± 0.47 [0.62 ± 0.48]^†^	0.61 ± 0.45 [0.63 ± 0.44]^†^
Stance phase (%)	68.19 ± 10.74	67.74 ± 8.15 [68.27 ± 9.13]^†^	69.14 ± 9.62 [68.53 ± 8.75]^†^
Swing phase (%)	31.81 ± 10.74	32.26 ± 8.15 [31.73 ± 9.13]^†^	30.86 ± 9.62 [31.47 ± 8.75]^†^
Maximum knee extension (°)	−1.02 ± 5.18	−2.88 ± 4.31 [−1.72 ± 3.74]^†^	−4.87 ± 4.34* [−2.74 ± 4.41]^†^
Dorsiflexion of the ankle at foot strike (°)	−8.91 ± 7.14	−6.93 ± 9.41 [−7.21 ± 7.17]^†^	−10.61 ± 7.92 [−7.97 ± 8.74]^†^
Maximum dorsiflexion of the ankle during the stance phase (°)	5.08 ± 8.73	6.68 ± 10.35 [5.70 ± 8.54]^†^	3.05 ± 8.13 [5.12 ± 7.52]^†^
Dorsiflexion of the ankle at the beginning of the swing phase (°)	−5.45 ± 3.11	−4.23 ± 4.95 [−5.32 ± 4.17]^†^	−7.18 ± 3.67 [−6.12 ± 3.97]^†^
Maximum plantar flexion of the ankle during the swing phase (°)	10.69 ± 5.11	10.99 ± 5.28 [10.72 ± 5.41]^†^	13.34 ± 4.75 [11.21 ± 6.12]^†^
Range of ankle motion (°)	16.58 ± 7.47	19.68 ± 5.41* [17.32 ± 6.54]^†^	17.28 ± 6.40 [17.97 ± 5.98]^†^

*Statistically significant difference (*t*-test, *P* < 0.05). Comparisons were performed between D0 and D9 (significant variation indicated in D9 column) and then between D0 and D31 (significant variation indicated in D31 column). No significant variation was identified between D9 and D31.

**Table 3:** Variation of electrophysiological ratios calculated for the soleus muscle and passive stiffness of the ankle (mean ± standard deviation) during the 1 Hz and 10 Hz († brackets) stimulation protocols: before (D0) and after (D9: early assessment; D31: late assessment).

Parameter	D0	D9	D31
*H*_max⁡_/*M*_max⁡_	0.75 ± 0.08	0.53 ± 0.08* [0.74 ± 0.07]^†^	0.49 ± 0.14* [0.75 ± 0.09]^†^
*T*/*M*_max⁡_	0.49 ± 0.09	0.33 ± 0.10* [0.48 ± 0.08]^†^	0.33 ± 0.16* [0.47 ± 0.09]^†^
Stiffness index (Nm/rad)	63.97 ± 21.09	61.62 ± 22.73* [63.54 ± 20.71]^†^	61.63 ± 22.39* [63.91 ± 21.41]^†^

*Statistically significant difference (*t*-test, *P* < 0.05). Comparisons were performed between D0 and D9 (significant variations indicated in D9 column) and then between D0 and D31 (significant variations indicated in D31 column). No significant variation was identified between D9 and D31.
